# Exercise accelerates recruitment of CD8^+^ T cell to promotes anti-tumor immunity in lung cancer via epinephrine

**DOI:** 10.1186/s12885-024-12224-7

**Published:** 2024-04-15

**Authors:** Sai-Nan Miao, Meng-Qi Chai, Xiang-Yu Liu, Cheng-Yu Wei, Cun-Cun Zhang, Ning-Ning Sun, Qing-Ze Fei, Lin-Lin Peng, Huan Qiu

**Affiliations:** School of Nursing, Anhui Medical University, 230032 Hefei, China

**Keywords:** Exercise, Lung cancer, Epinephrine, Ccl5, Cxcl10

## Abstract

**Background and purpose:**

In recent years, there has been extensive research on the role of exercise as an adjunctive therapy for cancer. However, the potential mechanisms underlying the anti-tumor therapy of exercise in lung cancer remain to be fully elucidated. As such, our study aims to confirm whether exercise-induced elevation of epinephrine can accelerate CD8^+^ T cell recruitment through modulation of chemokines and thus ultimately inhibit tumor progression.

**Method:**

C57BL/6 mice were subcutaneously inoculated with Lewis lung cancer cells (LLCs) to establish a subcutaneous tumor model. The tumor mice were randomly divided into different groups to performed a moderate-intensity exercise program on a treadmill for 5 consecutive days a week, 45 min a day. The blood samples and tumor tissues were collected after exercise for IHC, RT-qPCR, ELISA and Western blot. In addition, another group of mice received daily epinephrine treatment for two weeks (0.05 mg/mL, 200 µL i.p.) (EPI, *n* = 8) to replicate the effects of exercise on tumors in vivo. Lewis lung cancer cells were treated with different concentrations of epinephrine (0, 5, 10, 20 µM) to detect the effect of epinephrine on chemokine levels via ELISA and RT-qPCR.

**Results:**

This study reveals that both pre- and post-cancer exercise effectively impede the tumor progression. Exercise led to an increase in EPI levels and the infiltration of CD8^+^ T cell into the lung tumor. Exercise-induced elevation of EPI is involved in the regulation of Ccl5 and Cxcl10 levels further leading to enhanced CD8^+^ T cell infiltration and ultimately inhibiting tumor progression.

**Conclusion:**

Exercise training enhance the anti-tumor immunity of lung cancer individuals. These findings will provide valuable insights for the future application of exercise therapy in clinical practice.

**Supplementary Information:**

The online version contains supplementary material available at 10.1186/s12885-024-12224-7.

## Introduction

Lung cancer stands out as a prominent malignant tumor with a high global incidence [1]. According to GLOBOCAN 2020 data, there were approximately 2.21 million new cases of lung cancer in 2020, making it the second most prevalent cancer following breast cancer [2]. Lung cancer will account for about 11.4% of all diagnosed cancer cases and one in five deaths (18.0%) in 2020 [3, 4]. Lung cancer is primarily categorized into two histological subtypes: small cell lung cancer (SCLC) and non-small cell lung cancer (NSCLC), with NSCLC being the more prevalent form [5]. This poses a significant threat to human health, emerging as a substantial public health burden.

Currently, the treatment options for lung cancer include surgery, radiotherapy, chemotherapy, immunotherapy, and targeted therapy [6]. Accumulated research has documented that exercise therapy, as an adjuvant treatment for cancer, has garnered increased attention. Exercise is defined as a structured, repetitive, and purposeful physical activity aimed at improving health [7]. Appropriate exercise training has been shown to be safe, feasible, and effective in improving various outcomes in lung cancer patients [8, 9]. Studies indicate that exercise can reduce the risk of at least 13 different cancer types, with a 26% reduction in lung cancer, a 27% reduction in liver cancer, and a 42% reduction in esophageal cancer [10]. Exercise inhibits tumor growth and metastasis by regulating systemic factors such as immune function, signaling pathways, and cytokines [11]. However, the specific mechanisms of exercise intervention in lung cancer progression are not yet clear, and further research is still needed.

The tumor microenvironment (TME) plays a pivotal role in the process of tumorigenesis, with tumor-infiltrating lymphocytes (TILs) influencing the prognosis of the disease. TILs primarily consist of T lymphocytes, B lymphocytes, and natural killer (NK) cells [12]. Among them, local infiltrating CD8^+^ T cells emerge as the primary effector cells in anti-tumor immunity. Significantly, the number and distribution of infiltrating CD8^+^ T cells, both in the tumor parenchyma and stroma, have been reported to correlate with the survival period of tumor patients [13]. However, for CD8^+^ T cells to exert their tumor-killing effect, activation is essential. Exercise plays a role in increasing the number and activation of CD8^+^ T cells by regulating systemic metabolic factors and immune responses [14, 15]. CD8^+^ T cells further recognize and respond to tumor-associated antigens through T cell receptor (TCR), ultimately eliminating tumor cells [16]. A study in a mouse model of breast cancer has found that, an 8-week running intervention increased the ratio of intratumoral CD8^+^ T cells to Treg cells [17]. Another study confirmed that metabolic products derived from skeletal muscle during exercise, such as sodium lactate, can activate CD8^+^ T cells, thereby increasing cytotoxicity against targeted tumor cells [18]. Therefore, it becomes crucial to identify the key factors that facilitate the recruitment of effector T cells.

Exercise regulates the levels of numerous hormones in the body, with adrenaline being one of the most frequently affected hormones [19].Adrenaline is the main hormone in the adrenal medulla. The term “adrenaline” typically denotes the endogenous hormone, while “epinephrine” is commonly administered for medical purposes. In this study, both terms “adrenaline” and “epinephrine (EPI)” refer to the same molecule. It is well-established that exercise contributes to an elevation in serum EPI levels in the organism [20]. Some studies have revealed that the exercise-induced increase in EPI levels directly inhibits the activity of breast cancer cells and diminishes tumor formation in vivo by activating the tumor suppressor Hippo signaling pathway [21]. Additionally, EPI has the capability to selectively mobilize cells with cytotoxic effects, thereby promoting immune cell infiltration into tumors [22].

To investigate the anti-tumor mechanisms induced by exercise, we subcutaneously inoculated Lewis lung cancer cells (LLCs) into C57BL/6 mice to establish a xenograft mouse model. A treadmill-running exercise regimen was implemented in these mouse models of lung cancer. It is well known that the secretion of EPI increases after exercise. Additionally, exercise promotes the recruitment and activation of CD8^+^ T cells in tumors by driving Ccl5 and Cxcl10, thereby inducing anti-tumor immunity [23]. However, the underlying mechanism remains elusive. In this study, we demonstrated that exercise induces variations in Ccl5 and Cxcl10 levels through elevated EPI levels, subsequently increasing CD8^+^ T cell infiltration in mouse tumors. Additionally, EPI was utilized to treat a lung cancer subcutaneous mouse model, revealing anti-tumor effects that strikingly resemble those observed with exercise. Furthermore, EPI treatment effectively accelerated the secretion of Ccl5 and Cxcl10, promoting the recruitment of CD8^+^ T cells into the tumor. This research can provide recommendations for patients and even healthy individuals to establish suitable exercise plans in clinical practice and provide a reference for incorporating exercise into cancer treatment strategies.

## Materials and methods

### Animals

The animal study was approved by the Ethics Review Committee of Anhui Medical University approval (ethical approval No.84,230,009; No.84,230,012). All animal experiment schemes are carried out in accordance with the “animal experiment guide” and the study is reported in accordance with ARRIVE guidelines. C57BL/6 mice (22 ± 2 g), 8 weeks of age, from GemPharmatech. The animals were housed in the animal laboratory of Anhui Medical University, where the room temperature was 22 ± 2℃ and the relative humidity was 50–60%. 12-hour light and dark cycles were performed at a specific pathogen free barrier facility (Independent ventilation system, Suzhou, China). The xenografted tumor model was established by subcutaneously inoculating LLC cells (3 × 10^5^ cells per mouse) into the upper back of C57BL/6 mice. Tumor size was measured with a caliper every 2 days, and the tumor volume was determined with the formula: Length $\times$Width^2^$\times$ 0.52. After 2 weeks of exercise or free movement, all mice were sacrificed. All mice were euthanized using the cervical dislocation method.

### Cell culture

Lewis lung cancer (LLC) cells were kindly provided by Prof. Chenfeng Liu of the College of Life Sciences, Anhui Medical University. LLC cells were cultured in dulbecco’s modified eagle medium (DMEM) (Yeasen, 41401ES76) supplemented with 10% fetal bovine serum (Yeasen, 40130ES76), 100 IU/mL penicillin-streptomycin solution (Beyotime, C0222).

### Exercise intervention plan

We determined a moderate-intensity exercise intervention program to adapt to the characteristics of the mouse Lewis lung cancer model through literature search and exercise pilot experiment in mice [24]. The mice inoculated with subcutaneous tumor were randomly divided into different groups. The specific grouping is as follows: (1) Control (Ctrl): free movement for 8 weeks before inoculation and 2 weeks after inoculation tumor (*n* = 8); (2) Exercise-pre (Ex-pre): run for 8 weeks before inoculation tumor and free movement for 2 weeks after tumor inoculation (*n* = 8); (3) Exercise (Ex): run for 8 weeks before inoculation and 2 weeks after inoculation tumor (*n* = 8); (4) Exercise-post (Ex-post): free movement for 8 weeks before inoculation tumor and run for 2 weeks after inoculation tumor (*n* = 8). The Ex mice performed a moderate-intensity exercise program on a treadmill for 5 consecutive days a week, 45 min a day. The Ctrl mice was allowed free movement in the cage without exercise intervention. Use a treadmill (Shanghai Xinruan Information Technology, Shanghai, China) to calculate the daily running distance. Electrical stimulation (0.2 mA, 1 Hz) and sound and light stimulation were used to complete the daily exercise training amount of mice.

### Murine epinephrine treatment

The C57BL/6 mice inoculated with subcutaneous tumor were randomly divided into TC (*n* = 8), TE (*n* = 8), and EPI (*n* = 8). The TE mice underwent daily exercise sessions lasting 45 min for 14 consecutive days. EPI mice were injected daily with EPI (0.05 mg/mL, 200 µL i.p.) (Sigma E4642) for 14 consecutive days. For the acute effect of epinephrine[25], the mice were euthanized 20 min after injection of EPI on the 14th day using the cervical dislocation method, and blood, lung, and tumor tissue were collected.

### Sample collection

Due to the stress state of the mice just after exercise, within 24 h after the last exercise, pick the eyeball blood, let stand for 1–2 h at room temperature, and 3000 RPM centrifugal 10 min for serum samples, by ELISA detecting antigen antibody immune response. After the last blood collection, the mice were euthanized for cervical dislocation. The tumor was removed and weighed, measuring its volume as previously mentioned. The tissue was then divided into sections for the following analysis: (a) A lump of tumor was impended with formalin and paraffin wax prior to immunohistochemistry; (b) The second tumor tissue was refrigerated at -80 °C, and real-time quantitative polymerase chain reaction (RT-qPCR) was used to detect immune cell infiltration in the tumor.

### RNA sequencing (RNA-seq) analysis

RNA-seq was carried out by Seqhealth Technology Co., Ltd. (Wuhan, China). Total RNA was extracted from the tumor tissues of C57BL/6 mice with Ex or Ctrl mice. After RNA quality evaluation and library preparation, the library products were further sequenced with the Illumina NovaSeq 6000 sequencing platform. Differentially expressed genes (DEGs) were screened using thresholds of| log2 (fold change)| > 1 and *p* value < 0.05.

### Enzyme-linked immunosorbent assay (ELISA)

The mice were euthanized after exercise intervention using the cervical dislocation method. Blood samples were collected and centrifuged for serum collection. Epinephrine/Adrenaline ELISA kit (Cusabio, CSB-E08679m) was used to measure the levels of EPI concentration in sera according to the manufacturer’s instructions. The concentrations of Ccl5, Cxcl10 and Cxcl12 were measured in Lewis lung cancercellsculture supernatants and in the serum of mice using Ccl5 ELISA kit (Cusabio, CSB-E09256m), Cxcl10 ELISA kit (Cusabio, CSB-E08183m) and Cxcl12 (Cusabio, CSB-E04723m). The main ELISA kits were listed in Supplementary Table 1.

### Reverse-transcription quantitative polymerase chain reaction (RT-qPCR)

Total RNA was extracted from frozen tumor samples using TRIzol reagent (Invitrogen, 15,596). RNA concentration and quality were measured using a Spectrophotometer (DS-11, DeNovix). Reverse transcription was performed using MonScript™ RTIII Super Mix with dsDNase (Two-Step) (Monad, MR05201). RT-qPCR used MonAmp TM ChemoHS qPCR Mix (Monad, MQ00401) was employed to identify specific RNAs levels. RT-qPCR was performed on CFX96 Touch RT-qPCR Detection System (Bio-Rad, 785BR21049) under the following cycling conditions: 95 °C for 3 min, 40 cycles of 95 °C for 5 s, and 60 °C for 30 s, followed by the melting curve stage. Primers were synthesized by Beijing Tsingke Biotech Co., Ltd. β-actin served as internal references for mRNA. The mRNA levels were calculated using the 2^–ΔΔCq^ method. RT-qPCR was repeated at least three times. The primer sequences were shown in Supplementary Table 2.

### Western blot

Total proteins were isolated from LLC cells and mice tumors as previously described. Proteins were loaded and separated on 10% SDS-PAGE gels, concentrated on 5% SDS-PAGE gels, and then transferred onto a polyvinylidene fluoride (PVDF) membrane. The membrane was blocked with 10% non-fat dry milk (NFDM) /TBS-T (50 mM Tris, 1.37 mM NaCl, 2.7 mM KCl, 0.1% Tween 20; pH 7.4) buffer for 1 h and then incubated with diluted primary antibodies to PD-L1 (Proteintech, 66248-1-Ig, dilution 1:2000), P53 (Proteintech, 60283-2-Ig, dilution 1:1500) and GAPDH (Affinity, AF7021, dilution 1:2000) overnight at 4 °C. Membranes were washed four times with TBS-T buffer and incubated with horseradish peroxidase-conjugated secondary antibodies (Abmart, M21003/M21003, dilution 1:10000) for 1 h at room temperature. After washed five times with TBS-T, the bands were detected by using an ECL western blot substrate (Biosharp, BL520B). Finally, images were scanned and analyzed using a Hesper Chemiluminescence Imaging System (Monad, GD50401). The main antibodies applied in the western

blot assay were listed in Supplementary Table 1.

### Immunohistochemistry

Mouse tumor tissues were fixed with 4% paraformaldehyde. Paraffin-embedded sections of tumor were sliced to obtain 3 μm-thick tissue specimens. Tumor sections were deparaffinized with xylol and rehydrated with graded ethanol. Antigen retrieval was performed in antigen retrieval buffer. Endogenous peroxidase activity was blocked using 3% H_2_O_2_. Nonspecific protein interactions were blocked using 5% bovine serum albumin (BSA). Slides were incubated with primary antibodies at 4 °C overnight. The primary antibodies used are as follows: CD8 (Abcam, ab209775, dilution 1:1000), CD3 (Abcam, ab16669, dilution 1:1000), CD4 (Abcam, ab288724, dilution 1:1000), GZMB (Abcam, ab255598, dilution 1:1000), PD-L1 (Proteintech, 66248-1-Ig, dilution 1:1500), CD24 (Abcam, ab214231, dilution 1:500), CD56 (Proteintech, 14255-1-AP, dilution 1:10000) and CD11b (ABclonal, A1581, dilution 1:1500). Subsequently the sections were incubated with secondary antibodies (Vector, BA-1000, 1:300 dilution) and tertiary antibodies (Vector, PK-4000, 1:300 dilution) respectively for 30 min. Thereafter, incubation with DAB (Maxim, DAB-2031) was done and the sections were then counterstained with hematoxylin and examined by a light microscope. Specific staining was quantified using Image-Pro Plus 6.0 software. IHC staining density was evaluated and estimated based on the average staining intensity and the percentage of positively stained cells. The main antibodies used in the IHC assay were involved in Supplementary Table 1.

### Data analysis

All analysis data are presented as the mean ± SEM. Differences between the two or three groups were determined by two-tailed unpaired t tests or one-way analysis of variance (ANOVA). Statistical analysis was performed using SPSS 25.0 and GraphPad Prism 9.5.1. The *p* values < 0.05 were considered statistically significant.

## Results

### Exercise restricts lung tumor growth

**Fig. 1:**
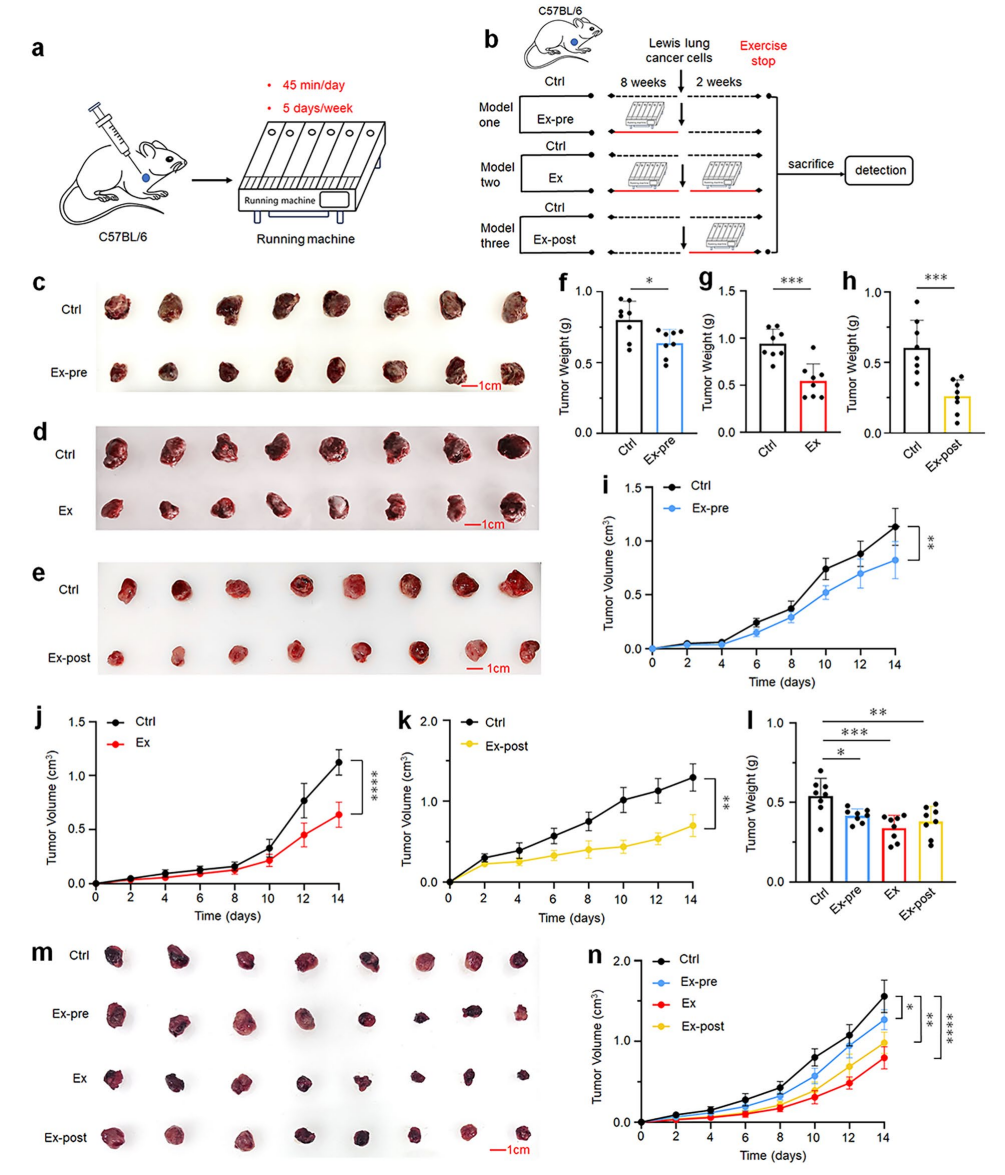
Exercise can delay tumor progression. **a** The forced-treadmill-running model (exercise) is illustrated. **b** Experimental grouping and design: Ctrl (control group), mice with free movement for 8 weeks before inoculation and 2 weeks after tumor inoculation (*n* = 8); Ex-pre (exercise before tumor inoculation), mice running for 8 weeks before tumor inoculation (*n* = 8); Ex: (exercise before and after tumor inoculation), mice that run for 8 weeks before inoculation and 2 weeks after tumor inoculation (*n* = 8); Ex-post (exercise after tumor inoculation), mice running for 2 weeks after tumor inoculation (*n* = 8). 45 min/day, 5 days/week. Solid red lines indicate exercise treatment, and dashed black lines indicate no exercise treatment. **c-e** The brightfield images display tumors from the three exercise models. **f** Comparison of tumor weight in the Ex-pre and Ctrl mice at the end of the exercise intervention (*n* = 8). **g** Comparison of tumor weight in the Ex and Ctrl mice at the end of the exercise intervention (*n* = 8). **h** Comparison of tumor weight in the Ex-post and Ctrl mice at the end of the exercise intervention (*n* = 8). **i-k** Mean of tumor volume changes after tumor formation in the three exercise models (*n* = 8). **l** Comparison of tumor weight in the Ctrl, Ex-pre, Ex and Ex-post mice at the end of the exercise intervention (*n* = 8). **m** The brightfield images display tumors from Ctrl, Ex-pre, Ex and Ex-post mice models. **n** Mean of tumor volume changes after tumor formation in the Ctrl, Ex-pre, Ex and Ex-post mice models (*n* = 8). Results of **f-k** are presented as mean ± SEM. **l** and **n** were performed using one-way analysis of variance (ANOVA). Statistical analysis was performed using two-tailed unpaired t tests. *p < 0.05, **p < 0.01, ***p < 0.001

To investigate the impact of exercise on lung tumor growth, the exercise intervened xenograft mouse model was established (Fig. 1a). The xenograft mouse model was created by subcutaneous injection with 100 µL (3 × 10^5^) of Lewis lung cancer cells (LLCs). Initially, the Ex-pre (exercise before tumor inoculation) model was established in this study. 16 mice were randomly assigned into two groups. The Ctrl mice (control group) were allowed to move freely in the cage for 8 weeks before LLCs injection and 2 weeks after LLCs injection. Ex-pre mice underwent a moderate-intensity exercise intervention regimen for 8 weeks pre-tumorigenesis and move freely in the cage for 2 weeks post-tumorigenesis. Exercise training was conducted for 45 min per day, 5 times per week (Fig. 1b). Furthermore, we evaluated the impact of running before tumor challenge in a subcutaneous lung cancer model in mice and observed that 8 weeks of pre-cancer exercise slowed tumor progression (Fig. 1c). As indicated, the weight and volume of tumors in the Ex-pre mice were lower than those in Ctrl mice (Fig. 1f, i).

Based on the above research, we further explored whether adding post-tumorigenesis exercise to pre-tumorigenesis exercise would have a more obvious tumor suppressive effect. Ex mice models were established. Another 16 mice were selected and randomly divided into two groups. We observed that 8 weeks of pre-tumorigenesis and 2 weeks of post-tumorigenesis exercise slowed significantly tumor progression (Fig. 1d). Moreover, the body weight and volume are significantly reduced after exercise, which can significantly inhibit tumor growth (Fig. 1g, j). In addition, to simulate cancer patients with different exercise habits, we further explored whether exercise after cancer has a favorable cancer suppression effect even without prior exercise before cancer. Therefore, we selected another 16 mice to construct post-tumorigenesis exercise model. Similarly, two weeks of running posterior to tumor cell inoculation could also significantly reduced tumor growth (Fig. 1e). As indicated, the weight and volume of tumors in the Ex-post mice (exercise after tumor inoculation) were significantly lower than those in Ctrl mice (Fig. 1h, k).

Furthermore, to compare group differences in tumor inhibition effects among the three exercise regimens, we re-selected four groups of mice for grouping intervention. The inhibitory effect of exercise in different model groups was consistent with the above results. All three exercise models showed tumor suppression (Fig. 1m). Among them, the inhibitory effect of Ex-pre mice was less. Interestingly, both Ex and Ex-post mice showed more significant tumor suppression. Compared with Ctrl mice, the Ex mice had a more pronounced inhibition of tumor growth than the Ex-post mice in terms of tumor weight and volume (Fig. 1l-n). Taken together, the above results vigorously suggested that moderate intensity exercise could significantly inhibit lung cancer tumor growth, no matter before or after tumorigenesis. Since the tumor suppression was better in the Ex and Ex-post mice, we will further explore the potential anti-tumor mechanism of exercise intervention in these two sets of mice models.

### Exercise promotes the infiltration of CD8^+^ T cell into the lung tumor

**Fig. 2:**
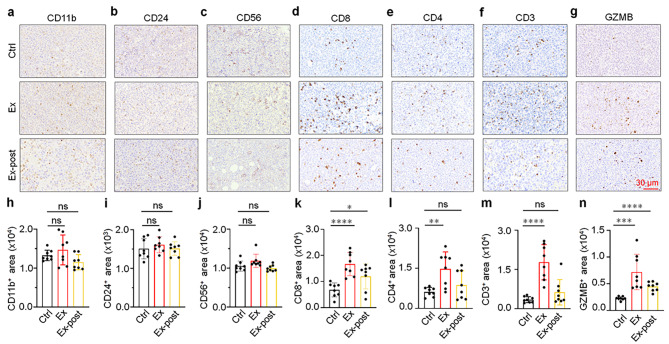
Exercise promotes infiltration of CD8, CD4, CD3^+^ T cell. **a-g** IHC staining of CD11b, CD24, CD56, CD8, CD4, CD3 and GZMB in the tumor in each group (*n* = 8). Scale bar: 30 μm. **h-n** Quantitative analysis of CD11b, CD24, CD56, CD8, CD4, CD3 and GZMB IHC staining in indicated groups (*n* = 8). The results of **h-n** are presented as mean ± SEM. Statistical analysis was performed using one-way analysis of variance (ANOVA). *p < 0.05, **p < 0.01, ***p < 0.001

Tumor development was closely associated with TME, and immune cells play a pivotal role in TME [26]. To further investigate the effect of exercise on tumor development, the potential anti-tumor mechanism was explored. Firstly, the infiltration of T cells (CD3, CD4, CD8, GZMB), tumor-associated macrophages (TAMs, CD11b), B cells (CD24) and NK cells (CD56) within the tumor was assessed by immunohistochemical (IHC) staining (Fig. 2a-g). The results show no statistical difference in the degree of TAMs (CD11b, marker of TAMs), B cells (CD24, marker of B cells), as well as NK cells (CD56, marker of NK cells) in tumor infiltration between the Ex and Ex-post models (Fig. 2h-j). However, the degree of CD8^+^ T cells infiltration in the tumors of Ex and Ex-post groups was higher than that in the Ctrl mice (Fig. 2d, k). Consistently, there were also significant differences in CD4^+^ and CD3^+^ T cells between the Ex and Ctrl mice (Fig. 2e-f, l-m). However, there was no significant difference in the degree of CD4^+^ and CD3^+^ T cells infiltration in tumor tissues between Ctrl and Ex-post mice (Fig. 2e-f, l-m).The expression of Granzyme B (GZMB), a T cell activation marker [27, 28] in both mouse models was higher than that in the Ctrl mice (Fig. 2g, n). Taken together, these studies have shown that exercise could promote the activation and infiltration of CD8^+^ T cells in lung adenocarcinoma tissues. In addition, as the exercise cycle of the Ex mice model is longer than that of the Ex-post model, the degree of T cell infiltration in the tumor is higher.

### Transcriptome analysis of mice tumor after exercise treatment

To better understand the characteristics and functions of T cells in tumor progression after exercise, we conducted an investigation into the transcriptome of tumors after exercise. Two mice were selected from the Ex and Ctrl groups, respectively, to explore how exercise affects signaling pathways and related gene expression.

**Fig. 3:**
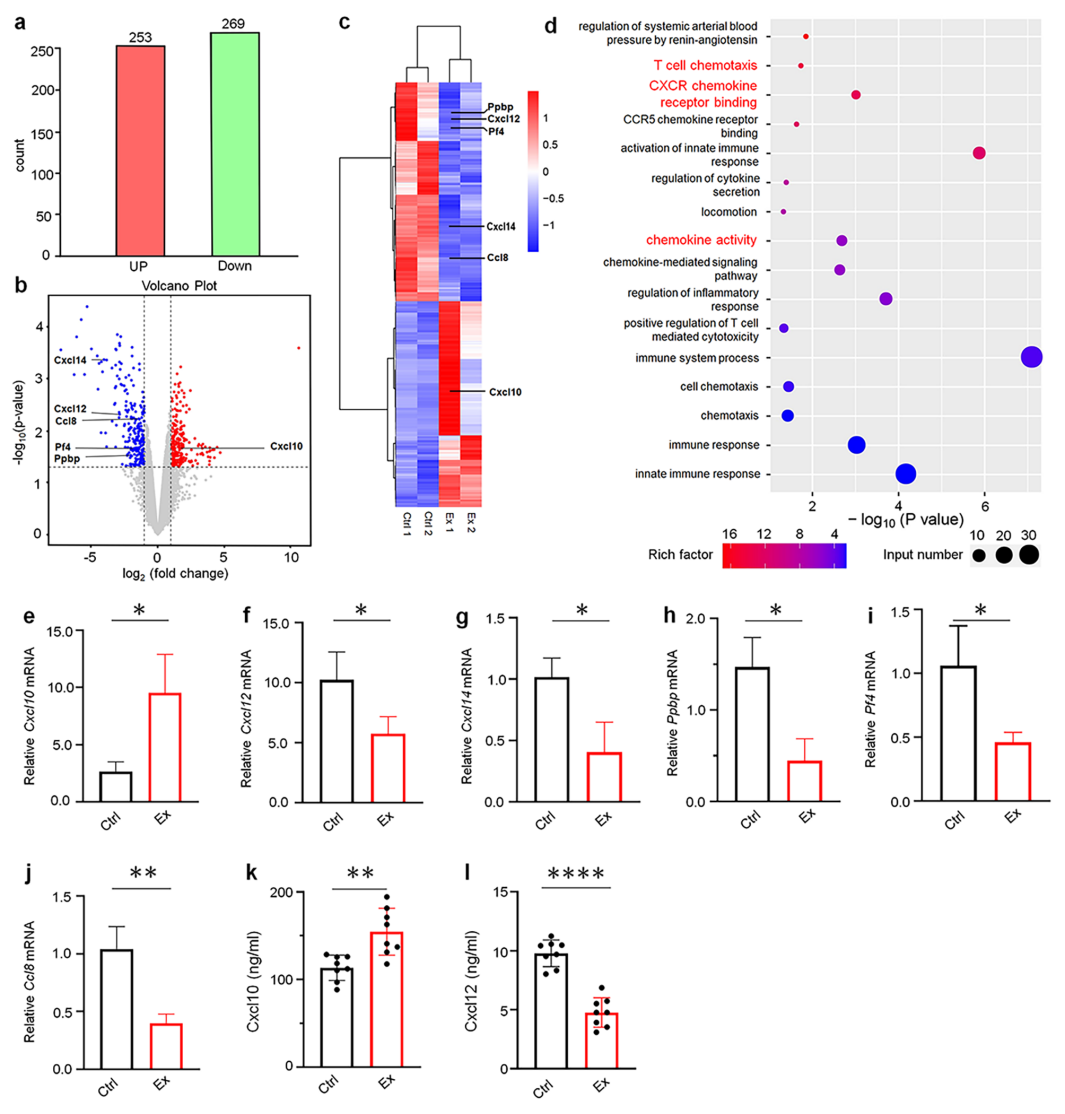
Transcriptional targets were examined by RNA-seq in tumors after exercise. **a** The number of differentially expressed genes in the Ex and Ctrl mice were detected by RNA-seq. **b** Volcano plot of differentially expressed genes in Ex compared with Ctrl tumor tissues. Red dots represent upregulated genes, and blue dots represent downregulated genes. **c** Heat map of the differentially expressed genes in Ex compared with Ctrl tumor tissues. **d** Pathway enrichment analysis of significantly upregulated or downregulated genes. **e-j** RT-qPCR validates the gene expression of *Cxcl10*, *Cxcl12*, *Cxcl14*, *Ppbp*, *Pf4* and *Ccl8* in the tumors each group (*n* = 3) after tumor inoculation. **k-l** The expression levels of Cxcl10 and Cxcl12 were detected in the tumors in each group by ELISA (*n* = 8). The results of **e-l** are presented as mean ± SEM. Statistical analysis was performed using two-tailed unpaired t tests. *p < 0.05, **p < 0.01, ***p < 0.001

A total of 522 differentially expressed genes (DEGs) were identified through a series of bioinformatics analyses, including 253 upregulated genes and 269 downregulated genes (Fig. 3a-b). A heat map of differential gene expression is displayed (Fig. 3c). The gene-hierarchical clustering of the expression profiles of each individual sample illustrated unique patterns of gene expression. Interestingly, the gene expression profiles between the Ex and Ctrl mice exhibited distinct patterns (Fig. 3c). To delve further into this phenomenon, enrichment analysis of Gene Ontology (GO) differentially expressed genes (DEGs) and KEGG pathways were performed. The DEGs enriched functions were screened in GO, revealing significant signaling pathways, including T-cell chemotaxis, chemokine activity, and CXCR chemokine receptor binding (Fig. 3d). A crucial role of *Cxcl10*, *Cxcl12*, *Cxcl14* and *Ccl8* for promoting regulatory T cells activation and proliferation [29–32]. PF4 levels negatively correlated with T cell function. Notably, *Ppbp* and *Pf4*, as key genes, play important roles in the prognosis and pathogenesis in lung adenocarcinoma (LUAD) [33, 34]. Meanwhile, *Cxcl10* has inhibitory effect on tumor progression, *Cxcl12*, *Cxcl14*, *Ppbp*, *Pf4* and *Ccl8* play an important role in promoting tumor progression [35–39]. Among them, *Cxcl10* was upregulated, while *Cxcl12*, *Cxcl14*, *Ppbp*, *Pf4* and *Ccl8* were downregulated after exercise (Fig. 3c). Furthermore, the above genes were verified in the Ex and Ctrl mice by RT-qPCR. Consistently, they all showed significant difference (*p* < 0.05) (Fig. 3e-j). To further verify whether exercise promotes T-cell chemotaxis, the expression of Cxcl10 and Cxcl12 in tumors of Ex and Ctrl mice was detected by ELISA, showing consistent evidence that exercise promotes the chemotaxis of Cxcl10 and Cxcl12 (Fig. 3k-l). Taken together, the above study suggests that exercise is conducive to T cell activity by up- or down-regulating the expression of related cytokines and chemokines.

### Exercise promote CD8^+^ T cell recruitment and mediate the anti-tumor effect by accelerate Ccl5 and Cxcl10 secretion

**Fig. 4:**
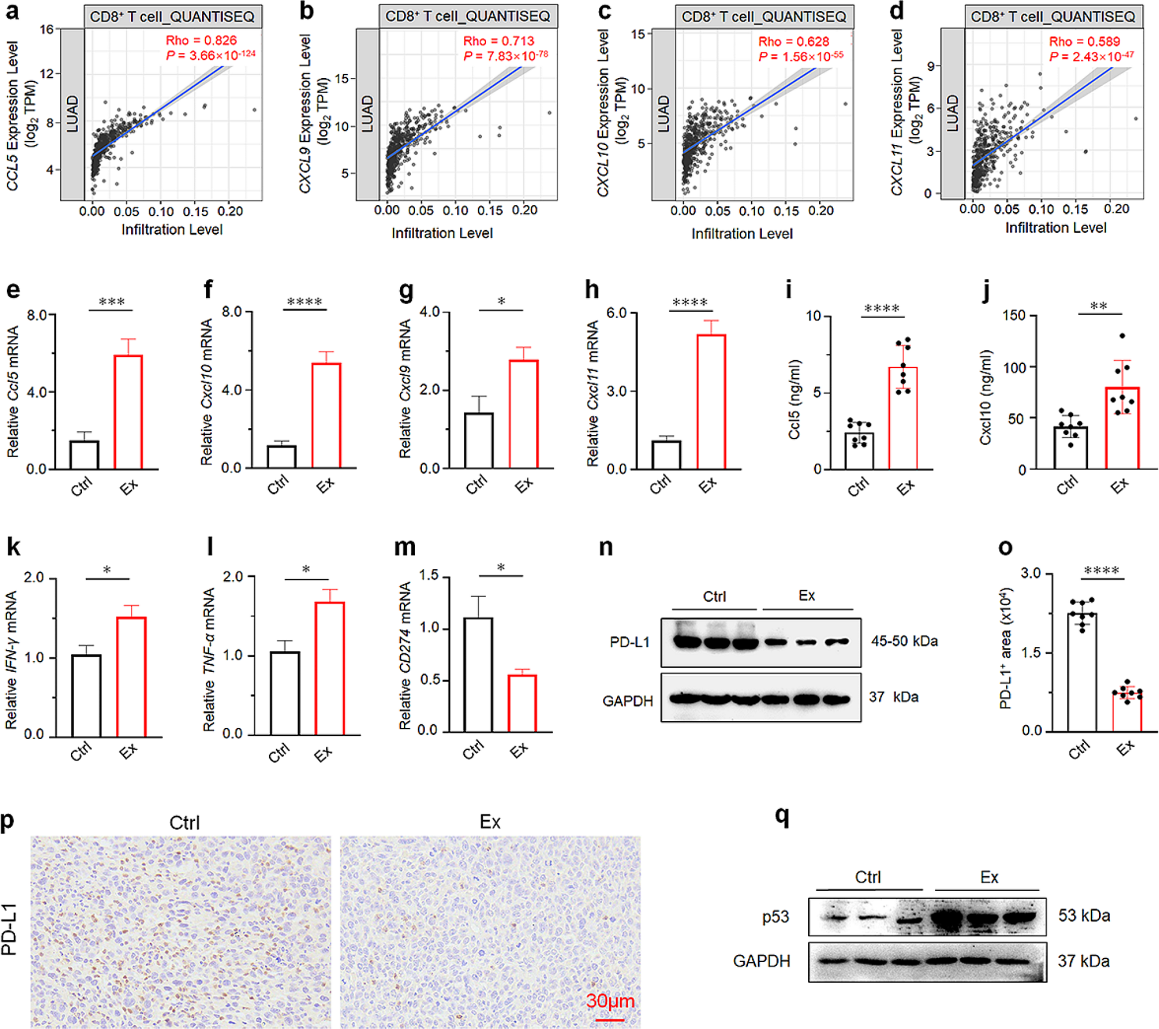
Exercise promotes CD8^+^ T cell recruitment with the assistance of Ccl5 and Cxcl10. **a-d** Correlation between Ccl5, Cxcl9, Cxcl10, and Cxcl11 mRNA level and CD8^+^ T cell using QUANTISEQ. Data were obtained from TIMER 2.0 (http://timer.comp-genomics.org/). **e-h** RT**-**qPCR analyses of mRNA expression of chemokines in the tumors of Ctrl and Ex groups (*n* = 3). **i-j** ELISA analysis of serum levels of Cxcl10 and Ccl5 in Ctrl and Ex mice (*n* = 8). **k-m** RT-qPCR analyses of mRNA expression of cytokines and PD-L1 in the tumors of Ctrl and Ex mice (*n* = 3). **n** Western blot analysis the expression of PD-L1 in the tumors of Ctrl and Ex mice (*n* = 3). Full-length blots/gels are presented in Fig. 4n of source data. **o** Automatic quantification of PD-L1 IHC staining in indicated mice tumors (*n* = 8). **p** IHC staining of PD-L1 on tumor sections from different groups (*n* = 8). **q** Western blot analysis the expression of P53 in the tumors of Ctrl and Ex mice (*n* = 3). Full-length blots/gels are presented in Fig. 4q of source data. The results of **e-m** and **o** are presented as mean ± SEM. Statistical analysis was performed using two-tailed unpaired t tests. *p < 0.05, **p < 0.01, ***p < 0.001

Next, we attempted to investigate the key factors that affect CD8^+^ T cell infiltration after exercise treatment. Chemokines, small cytokines or signaling proteins secreted by tumor cells, stromal cells, and immune cells, play a crucial role in CD8^+^ T cell infiltration [40]. Cxcr3, a chemokine receptor with anti-tumor effects expressed in effector CD8^+^ T cells, Th1 cells, and NK cells, interacts with its ligands Cxcl9, Cxcl10, and Cxcl11, promoting immune cell recruitment to TME [41, 42]. Utilizing TIMER 2.0, we evaluated the association between chemokine mRNA levels and CD8^+^ T cells through various algorithms. As summarized in Fig. 4a-d, the mRNA levels of Ccl5, Cxcl10, Cxcl9, and Cxcl11 positively correlated with CD8^+^ T cells infiltration. RT-qPCR was employed to detect the mRNA levels of these chemokines in tumor tissues of our exercise models, confirming that Ccl5, Cxcl10, Cxcl9, and Cxcl11 were significantly increased in Ex mice compared to the Ctrl group (Fig. 4e-h). Overall, these data suggest that exercise promotes the expression of Ccl5, Cxcl10, Cxcl9, and Cxcl11, further promoting the recruitment of CD8^+^ T cells. Specifically, the expression of Ccl5 and Cxcl10 is dramatically higher compared to other chemokines. Furthermore, Ccl5 and Cxcl10 ELISA date showed that both the Ccl5 and Cxcl10 in the Ex mice were up-regulated compared with the Ctrl group (Fig. 4i-j).

On the other hand, T cell infiltration also requires the help of cytokines. Such as IFN-γ and TNF-α, both produced by NK cells and activated T cells, further enhancing the proliferation of T cells and slowing down the proliferation of cancer cells [43–45]. The ability of T cells to express IFN-γ and TNF-α plays an important role in anti-tumor immune response [46]. In our model, both IFN-γ and TNF-α were upregulated after exercise (Fig. 4k-l). The results showed that T cell function was enhanced by exercise in xenograft tumors. PD-L1/*CD274*, a protein on the surface of tumors that binds to PD-1 on the surface of T cells to weaken effector T cell responses, further induces immune suppression in tumor environments [47]. A study on SCLC therapy has demonstrated that DNA damage response (DDR) inhibitors suppress PD-L1-induced immunosuppression by enhancing the expression of Cxcl10 and Ccl5 in the STING/TBK1/IRF3 pathway [48]. Suggesting that chemokines are involved in regulating PD-L1 levels. Our findings show that exercise is involved in regulating chemokine levels (Fig. 4e-j). Therefore, we questioned whether exercise regulates the level of PD-L1. RT-qPCR analysis revealed that exercise significantly decreased the expression of PD-L1 in tumors (Fig. 4m). Furthermore, we assessed differences in PD-L1 protein levels between Ex and Ctrl mice. Coherently, the expression of PD-L1 was significantly lower in Ex mice (Fig. 4n). The IHC results indicated that the tumors from Ex mice were predominantly negative for PD-L1 compared to Ctrl mice (Fig. 4o-p). Research reveals that local activation of p53 also results in reversing immunosuppression and enhancing antitumor immunity in TME [49]. Next, we wanted to clarity whether exercise could modulate p53, thus p53 protein were detected between Ex and Ctrl mice. Interestingly, the expression of p53 was significantly higher in Ex mice (Fig. 4q). Hence, we infer that exercise could also act on TME by regulating p53. Collectively, the above results suggested that exercise could activate anti-tumor immunity to inhibit tumor growth by multiple pathways, such as inducing chemokines and cytokines especially Ccl5 and Cxcl10, to promote CD8^**+**^ T cell recruitment, and regulating the expression of PD-L1 and p53 to mediate the anti-tumor effect.

### Exercise inhibits tumor progression by upregulation of EPI

**Fig. 5:**
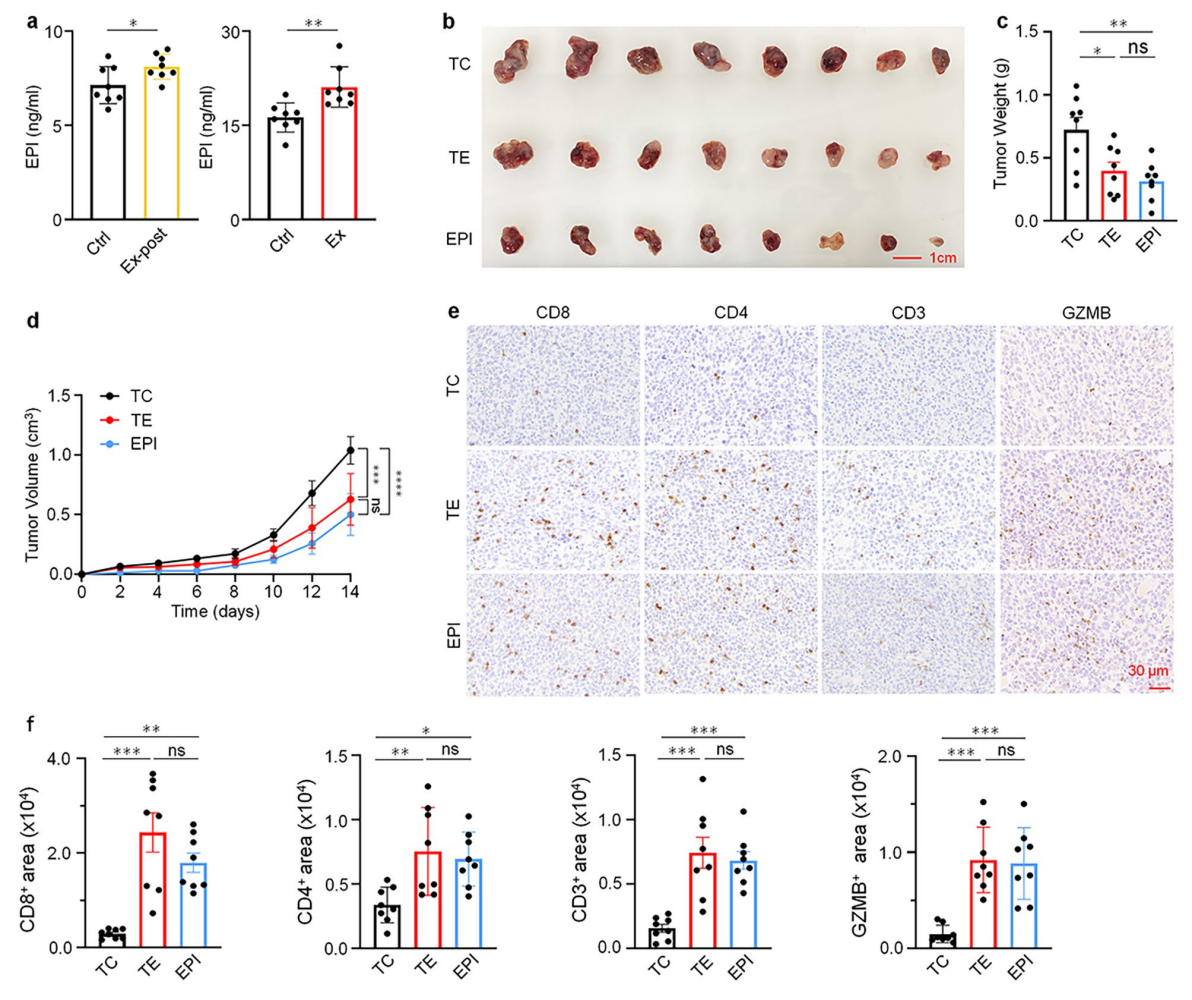
Exercise inhibits tumor progression by upregulation of EPI levels. **a** Serum levels of EPI in Ctrl, Ex and Ex-post groups were detected by ELISA in tumor-bearing mice (*n* = 8). **b-d** EPI mice receiving daily injections of EPI (0.5 mg/kg i.p.) (*n* = 8) and TE mice with daily running for 14 days after tumor inoculation (*n* = 8) compared with TC mice (*n* = 8). **b** Representative images. **c** Tumor weights of indicated groups. **d** Tumor volumes of different groups. **e** IHC staining of CD8, CD4, CD3 and GZMB on tumor sections from indicated groups (*n* = 8). **f** Automatic quantification of CD8, CD4, CD3 and GZMB IHC staining in indicated mice tumors (*n* = 8). Data are presented as the mean ± SEM in each group. Statistical analysis of **a** was performed using two-tailed unpaired t tests. **c**, **d** and **f** were performed using one-way analysis of variance (ANOVA). *p < 0.05, **p < 0.01, ***p < 0.001

Next, we aim to uncover the precise mechanism by which exercise enhances anti-tumor immunity. Moderate exercise activates the adrenal glands of the sympathetic nervous system, leading to the secretion of epinephrine, which activates the heart and muscles to prepare for the “fight or escape” response [50, 51]. In addition, epinephrine can also improve skeletal muscle performance by promoting the breakdown of muscle glycogen and the utilization of glucose [52]. Previous studies have suggested that EPI regulates tumors by influencing the immune system [53, 54], but the underlying mechanism needs further investigation. To explore the suppressive function of EPI in carcinogenesis, serum EPI levels were validated, and the results showed an elevation in EPI abundance in both exercise groups (Fig. 5a).

Furthermore, to investigate whether EPI could replicate the effects of exercise on tumors in vivo, a new batch of tumor-bearing mice were randomly assigned to three groups, the tumor control group (TC, *n* = 8), the tumor exercise group (TE, *n* = 8) and the EPI group. The TE mice exercised for 14 days after tumor inoculation, while the EPI group mice received daily EPI treatment for 14 days (0.05 mg/mL, 200 µL i.p.). The result demonstrated that EPI injection also led to a reduction in tumor weight and volume. However, there was no significant difference in the anti-tumor effects between exercise and EPI treatment (Fig. 5b-d). To elucidate whether EPI correlates with T cell activation in tumor-bearing mice, we performed IHC staining for CD8^**+**^, CD4^**+**^ and CD3^**+**^ T cells in the tumors of three groups. Furthermore, GZMB was also examined. The IHC result showed that the infiltration of CD8^**+**^, CD4^**+**^, CD3^**+**^ T cells and the expression of GZMB were obviously increased in both EPI and TE groups (Fig. 5e-f). Strikingly, the number of CD8^**+**^ T cell infiltrating is higher than CD4^**+**^ and CD3^**+**^ T cell. The number of infiltrated CD4^+^ cells was about 69.1% lower than that of CD8^+^ T cells, and the number of CD3^+^ cells was about 69.5% lower (Fig. 5e-f). Altogether, these findings demonstrated that exercise-induced increased serum EPI levels in mice, further promotes T cell activation and inhibit tumor growth in vivo.

### Exercise-induced tumor suppression by upregulation of Ccl5 and Cxcl10 in the tumor microenvironment via elevation of EPI

**Fig. 6:**
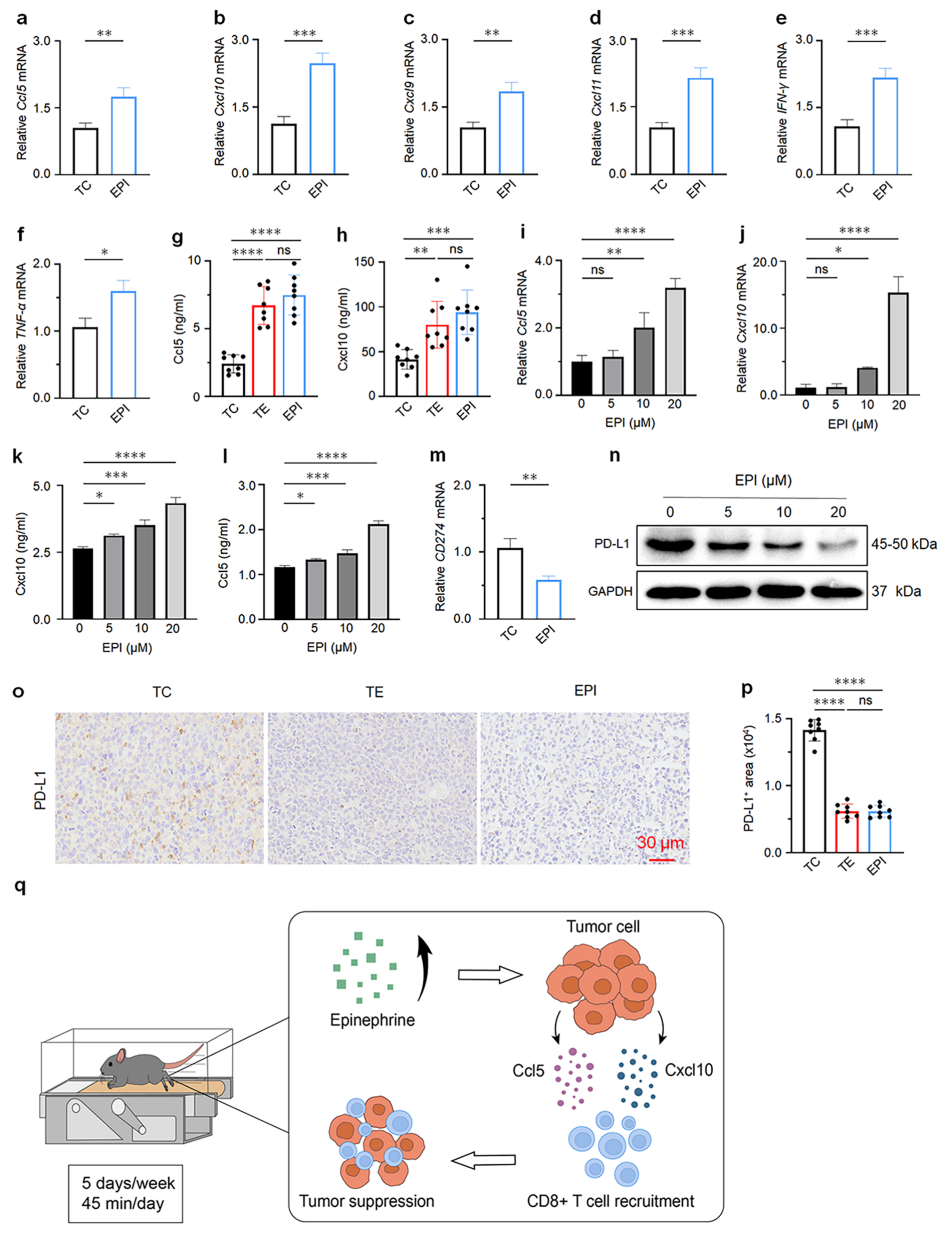
EPI regulates changes in chemokine levels. **a-f** RT-qPCR analyses the mRNA expression of chemokines and cytokines in the tumors of TC and EPI mice (*n* = 3). **g-h** ELISA analysis serum levels of Cxcl10 and Ccl5 in TC, TE and EPI treated mice (*n* = 8). **i-j** RT-qPCR detect the mRNA levels of Cxcl10 and Ccl5 in LLC cells after treated with different concentrations of EPI (0, 5, 10, 20 µM). **k-l** Cxcl10 and Ccl5 in the supernatant of LLC cells were measured by ELISA. **m** RT-qPCR analyses the mRNA expression of CD274 in the tumors of control TC and EPI groups (*n* = 3). **n** Western blot analyses PD-L1 in LLC cells after EPI treatment for 24 h at different concentration (0, 5, 10, 20 µM). Full-length blots/gels are presented in Fig. 6n of source data. **o** IHC staining of PD-L1 on tumor sections from indicated groups (*n* = 8). **p** Automatic quantification of PD-L1 IHC staining in indicated mice tumors (*n* = 8). **q** Graphical abstract of this study. Exercise-induced elevation of EPI is involved in regulating Ccl5 and Cxcl10 expression, subsequently promoting CD8^**+**^ T cells recruitment, and ultimately inhibiting tumor progression. Data are presented as the mean ± SEM in each group. Statistical analysis of **a-f** and **m** were performed using two-tailed unpaired t tests. **g-l** and **p** were performed using one-way analysis of variance (ANOVA). *p < 0.05, **p < 0.01, ***p < 0.001

To understand how the surge of EPI induces T cell activation by exercise, we examined the chemokines associated with T cell recruitment. RT-qPCR analysis was conducted on tumor-bearing mice after EPI injection, mirroring the results discussed above in result 4 (Fig. 4e-h, k-l). The expression of chemokines (Ccl5, Cxcl9, Cxcl10, Cxcl11) and cytokines (IFN-γ, TNF-α) increased after EPI treatment (Fig. 6a-f). Given the significant increase in mRNA levels of Cxcl10 and Ccl5 compared to other chemokines in the previous results (Fig. 4e-f), we further detected the expression of Ccl5 and Cxcl10 in tumors of the EPI groups using ELISA. Consistently, both Ccl5 and Cxcl10 in the EPI group were upregulated, compared to the TC group (Fig. 6g-h).

Furthermore, we explored whether EPI could regulate chemokine levels. LLCs were treated with different concentrations of EPI (0, 5, 10, 20 µM). After 24 h of drug treatment, RT-qPCR analysis was performed on the four groups. The results showed that compared with the control group (0 µM), the expression of Ccl5 and Cxcl10 in LLC cells significantly increased after EPI treatment in a concentration-dependent manner (Fig. 6i-j). In addition, Ccl5 and Cxcl10 in supernatant of LLC cells were also detected after EPI treatment through ELISA assay, showing an increase in a concentration-dependent manner (Fig. 6k-l). It indicated that EPI is involved in regulating the expression of Ccl5 and Cxcl10.

Consistent with the above finding that exercise can affect the mRNA level of PD-L1 in Ex mice tumors (Fig. 4m), we found that EPI could also clearly diminish the mRNA expression of PD-L1 in vivo (Fig. 6m). Simultaneously, protein was extracted from LLCs after EPI treatment for 24 h at different concentrations, and the expression of PD-L1 was performed by western blot. The results showed that the expression of PD-L1 in EPI-treated cells was also decreased in a concentration-dependent manner (Fig. 6n). Consistent with this, EPI substantially reduced PD-L1 expression in vivo (Fig. 6o-p). In summary, these results suggested that exercise- induced elevation of EPI and inhibition of the expression of PD-L1 protein upregulate the level of Ccl5 and Cxcl10, promoting the recruitment of CD8^+^ T cells and subsequently suppressing the development of lung tumor (Fig. 6q).

## Discussion

This study focused on exercise and link exercise, cancer, and immunity, which identified a potential relationship between exercise-induced EPI and the expression of chemokines that may restrict tumor growth in lung cancer-bearing mice and cell lines. In mice with lung cancer, exercise was observed to alter the TME. Additionally, exercise led to an increase in EPI levels and the recruitment of CD8^+^ T cells. Finally, we explored the mechanism by which exercise inhibited tumor progression by inducing EPI that promoting Ccl5 and Cxcl10 release from tumor cells further leading to enhanced T lymphocyte infiltration (Fig. 6q).

Studies have extensively investigated exercise training as an adjunct to cancer treatment, demonstrating unprecedented clinical efficacy [55, 56]. Aerobic exercise training mitigates tumor growth and cancer-induced splenomegaly through modulation of non-platelet platelet factor 4 expression, and reduces the incidence of several cancers [57]. Furthermore, exercise promotes immune mobilization and accumulation of tumor-infiltrating IL-15Rα^+^ CD8 T cells, which reduces tumor growth effectively [20]. In mice, CD8^+^ T cells are metabolically altered by exercise in a manner that acts to improve their antitumoral efficacy and inhibit of tumor growth [58]. Our evidences demonstrated that exercise enhanced the degree of T cell infiltration into the tumor, especially CD8^+^ T cells. However, high-intensity training reduces CD8^+^ T cell redistribution in response to exercise, which may imply reduce immune surveillance in human [59]. Therefore, we use a moderate-intensity exercise intervention program, which had a certain tumor suppression effect. However, the molecular mechanisms underlying the association between exercise training and anti-lung cancer effects are poorly understood. Deciphering the molecular regulatory mechanisms of exercise training is crucial for advancing cancer therapy.

Notably, exercise has been found to mobilize NK cells through EPI. A surge in exercise-related EPI, achieved through daily low-dose EPI injections, resulted in a remarkable 61% reduction in tumor volume [60]. In a previous study, Hojman et al. reveal that exercise decreases tumor incidence and growth by over 60% across several mouse tumor models through a direct regulation of NK cell mobilization and trafficking in an epinephrine and IL-6 dependent manner [25]. Furthermore, EPI significantly inhibited the in vitro and in vivo proliferation of LNCaP95 prostate cancer cells, which are as a promising therapeutic agent to treat cancer [61]. In particular, modulation of anti-tumor immunity by the adrenergic system was reported for β2-adrenergic signaling [62]. Yet, the role of EPI in cancer is complex. YAP is an oncoproteins of Hippo tumor suppressor signaling pathway, are associated with tumor growth and metastasis [21]. According to a report, in MDA-MB-231 breast cancer cells, EPI stimulation has been shown to resulted in phosphorylation of YAP and thus inactivated [63]. During exercise, a surge of EPI can mobilize CD8^+^ T cells. CD8^+^ T cells are a specific type of T cells known for their killing effect on antigen substances, including certain viruses and tumor cells, making them a crucial component of the body’s anti-tumor response [18]. In a previous research, T cells with an activated phenotype which were mobilized after EPI stimulation, especially CD8^+^ T cells [64]. Meanwhile, EPI augments antigen-specific T cell immune responses in C57BL/6 mice by a CD8^+^ T cell-dependent mechanism [65]. We confirmed in vitro that exercise can elevate EPI levels and lead to changes in various chemokines and cytokines, with Ccl5 and Cxcl10 showing particularly pronounced elevation. Concurrently, the increased expression of these chemokines facilitated the recruitment of CD8^+^ T cells. Targeting β2 adrenergic receptors inhibits human T cell function either directly or indirectly, with a stronger suppressive effect on CD8 than on CD4^+^ T cells [66]. Our findings suggest that EPI induced changes in the tumor-immune milieu both in vivo and in vitro. Elevated EPI levels can notably promote the release of chemokines from tumor cells and recruit CD8^+^ T cells, thereby inhibiting tumor progression, aligning with the observed effects of exercise. Simultaneously, PD-L1, an important immunosuppressive factor that interacts with the PD-1 receptor to inhibit T cell activation [67–69]. Moreover, abnormally high PD-L1 expression on tumor cells in TME mediates tumor immune escape, and the development of anti-PD-1/PD-L1 antibodies has recently become a hot topic in cancer immunotherapy [70]. Our research showed PD-L1 reduced expression in response to exercise-induced epinephrine elevation. This suppression of PD-L1 further promotes CD8^+^ T cell recruitment, ultimately leading to the inhibition of tumor progression.

Studies have shown that key genes may be potential biomarkers for improved prognosis and treatment of lung cancer to reveal the composition of different cell types and functions [71–73]. RNA sequencing analysis revealed training-induced upregulation of pathways associated with immune function. We observed that exercise has the potential to promote T cell chemotaxis, up-regulate *Cxcl10* and down-regulate *Cxcl12* expression. Chemokines, characterized as small cytokines or signaling proteins, can be secreted by tumor cells to recruit CD8^+^ T cells to the tumor site, influencing tumor progression [74, 75]. Prior studies have demonstrated that CD8^+^ T cells undergo functional recruitment in tumor tissues, with corresponding upregulation of chemokine receptors on human CD8^+^ T cells. Various chemokines can also stimulate the migration of CD8^+^ T cells [74]. Cxcr3 is a chemokine receptor with Cxcl9, Cxcl10, and Cxcl11. One of these chemokines, Cxcl10, not only attracts CD8^+^ effector T cells to sites of inflammation, but also direct their polarization into highly potent effector T cells, to restrain tumor growth and enhance anti-tumor immunity [76]. Previous research has shown that inhibiting KDM4C induces the transcription of Cxcl10, enhancing CD8^+^ T cell-mediated antitumor immune responses in lung cancer [14]. In addition, chemokine Ccl2 and Cxcl10 induction can be blocked by voluntary exercise, ameliorating a proinflammatory phenotype within the prefrontal cortex of mice fed a western diet [77]. Moreover, Ccl5 production in lung cancer cells can impact the immune microenvironment. High Ccl5 expression is associated with poor prognosis and diminished CD8 effector function in lung cancer patients [78]. Ccl5 production in lung cancer cells contributes to the immunosuppressive lung environment, promoting tumor development. Our results emphasize that elevated EPI promotes Ccl5 and Cxcl10 recruitment of CD8^+^ T cells to the tumor site, thereby inhibiting tumor progression in an exercise-induced lung cancer model. Therefore, chemokines play a vital role in T cell function [79].

Some innovative aspects are evident in this study. Our research demonstrates that exercise inhibits tumor progression by promoting the secretion of EPI and inhibiting the expression of PD-L1, while also enhancing the secretion of T cell-associated chemokines and activating T cells. Notably, exercise induces changes in chemokine levels, promoting the infiltration of CD8^+^ T cells. Additionally, the inhibitory effect of exercise on tumor was validated using three different exercise regimens, providing valuable insights for the improved application of exercise therapy to clinical practice. Furthermore, understanding the mechanism of exercise-induced changes to the tumor-immune milieu can offer new perspectives to enhance patients’ adherence to exercise in the future. However, this study has limitations, such as a relatively singular model, and the specific regulatory mechanism of exercise on PD-L1 is not deeply explored in this paper. Additionally, the exercise intervention protocol involved mice in experiments but did not involve human studies or combinations with other treatment options. Future work will be necessary to determine the clinical feasibility of rigorous exercise regimens for patients with lung cancer. Exercise intervention is a promising strategy for cancer treatment. In the future, we will continue to explore the deep mechanism of exercise inhibiting the development of lung cancer and provide new ideas for the precise treatment of lung cancer.

## Conclusion

In conclusion, our study revealed that exercise could inhibit tumor onset and progression by promoting the release of adrenaline and harmonizing chemokines such as Ccl5 and Cxcl10. Furthermore, it enhances the infiltration of CD8^+^ T cells, thereby bolstering anti-tumor immunity. This research delved into the impact of exercise therapy on tumor progression, considering three different models of exercise. This approach aims to broaden awareness about the benefits of exercise as an adjuvant therapy for tumor treatment. Ultimately, our findings provide valuable insights for the future application of exercise therapy in clinical practice.

### Electronic supplementary material

Below is the link to the electronic supplementary material.

Supplementary Material 1

Supplementary Material 2

Supplementary Material 3

Supplementary Material 4

Supplementary Material 5

Supplementary Material 6

## Data Availability

The datasets used and/or analyzed during the current study available from the corresponding author on reasonable request.

## Abbreviations

LLCs: Lewis lung cancer cells
SCLC: Small cell lung cancer
NSCLC: Non-small cell lung cancer
TME: Tumor microenvironment
TILs: Tumor-infiltrating lymphocytes
NK: Natural killer cells
EPI: Epinephrine
DMEM: Dulbecco’s modified eagle medium
GZMB: Granzyme B
DEGs: Differentially expressed genes
GO: Gene Ontology
DEGs: Differentially expressed genes
LUAD: Lung adenocarcinoma
DDR: DNA damage response
