# Supplementary material for: Exercise accelerates recruitment of CD8+ T cell to promotes anti-tumor immunity in lung cancer via epinephrine
Source: BMC Cancer. 2024 Apr 15;24:474. doi: 10.1186/s12885-024-12224-7 (PMC11021002; doi:10.1186/s12885-024-12224-7)
Supplement: Supplementary file 1 — Supplementary Material 1 [file 12885_2024_12224_MOESM1_ESM.pdf]

Supplementary Tables

Supplementary Table 1. Key resources table

Supplementary Table 2. The primer sequences used for RT-qPCR

Supplementary Table 1. Key resources table

REAGENT or RESOURCE	SOURCE	IDENTIFIER
**Antibodies**		
**Antibodies/IHC**		
CD8	Abcam	ab209775; RRID: AB_2860566
CD3	Abcam	ab16669; RRID: AB_443425
CD4	Abcam	ab288724; RRID: AB_2941893
GZMB	Abcam	ab255598; RRID: AB_2860567
CD11b	Abclonal	A1581; RRID: AB_2763232
CD24	Abcam	ab214231; RRID: AB_10123186
CD56	Proteintech	14255-1-AP; RRID: AB_2149421
**Antibodies/WB**		
GAPDH	Affinity	AF7021; RRID: AB_2839421
PD-L1	Proteintech	66248-1-Ig; RRID: AB_2839421
P53	Proteintech	60283-2-Ig; RRID: AB_2881401
Goat Anti-Rabbit & Mouse IgG-HRP	Abmart	M21003; RRID: AB_2920649
Goat Anti-Mouse IgG HRP	Abmart	M21001; RRID: AB_2713950
**Chemicals, Peptides, and Recombinant Proteins**		
Epinephrine	Sigma	E4642
**Critical Commercial Assays**		
RT-qPCR kit	Monad, China	MR05201
Goat Anti-Rabbit IgG	Vector Laboratories	BA-1000
Equine Anti-mouse IgG	ZSGB-BIO, China	ZB-2020
VECTASTAIN® ABC-HRP Kit	Vector Laboratories	PK-4000

DAB staining kit	Maxim, China	2031
**ELISA Kit**		
Mouse Epinephrine/Adrenaline (EPI)	Cusabio, China	CSB-E08679m
Mouse regulated on activation in normal T-cell expressed and secreted (CCL5)	Cusabio, China	CSB-E09256m
Mouse interferon-inducible protein 10 (CXCL10)	Cusabio, China	CSB-E08183m
Mouse Stromal cell derived factor 1β (CXCL12)	Cusabio, China	CSB-E04723m
Cell counting kit-8	Raymond Cobo, China	RK001099

Supplementary Table 2. The primer sequences used for RT-qPCR

Gene	Sequence
M-*β-actin*	Forward: 5′-CTAAGGCCAACCGTGAAAAG-3′
	Reverse: 5′-ACCAGAGGCATACAGGGCA-3′
M-*Ccl5*	Forward: 5′-GTGCCCACGTCAAGGAGTAT-3′
	Reverse: 5′-TCGAGTGACAAACACGACTG-3′
M-*Cxcl9*	Forward: 5′-CTCGGACTTCACTCCAACACA-3′
	Reverse: 5′-ATCACTAGGGTTCCTCGAACT-3′
M-*Cxcl10*	Forward: 5′-GCTCAGGCTCGTCAGTTCTAAGT-3′
	Reverse: 5′-GGAAGATGGTGGTTAAGTTCGTC-3′
M-*Cxcl11*	Forward: 5′-GTAACGGCTGCGACAAAGTTGAAG-3′
	Reverse: 5′-GAGGCGAGCTTGCTTGGATCTG-3′
M-*CD274*	Forward: 5′-CCATACCGCAAAATCAACCAG-3′
	Reverse: 5′-GACACTTCTCTTCCCACTCAC-3′
M-*IFN-γ*	Forward: 5′-AGGAACTGGCAAAAGGATGGT-3′
	Reverse: 5′-TCATTGAATGCTTGGCGCTG-3′
M-*TNF-α*	Forward: 5′-GTTGTACCTTGTCTACTCCCAG-3′
	Reverse: 5′-GGTTGACTTTCTCCTGGTATGAG-3′
M-*Cxcl12*	Forward: 5′-CCTTCAGATTGTTGCACGGC-3′
	Reverse: 5′-TACCGTCAGGTTTGAGCACC-3′
M-*Cxcl14*	Forward: 5′-AGCCAAAGTACCCACACTGC-3′
	Reverse: 5′-TCTTCGTAGACCCTGCGCT-3′
M-*Ppbp*	Forward: 5′-TGCTGATGTGGAAGTGATAGCC-3′
	Reverse: 5′-AGCAGCTGGTCAGTAACCTTC-3′
M-*Pf4*	Forward: 5′-AGAGCCCTAGACCCATTTCCT-3′
	Reverse: 5′-CATTCTTCAGGGTGGCTATGAG-3′
M-*Ccl8*	Forward: 5′-CTACGCAGTGCTTCTTTGCC-3′
	Reverse: 5′-GGTGACTGGAGCCTTATCTGG-3′
