# Supplementary figures and images for: Exercise accelerates recruitment of CD8+ T cell to promotes anti-tumor immunity in lung cancer via epinephrine
Source: BMC Cancer. 2024 Apr 15;24:474. doi: 10.1186/s12885-024-12224-7 (PMC11021002; doi:10.1186/s12885-024-12224-7)

## Original images for WB

Figure 4n

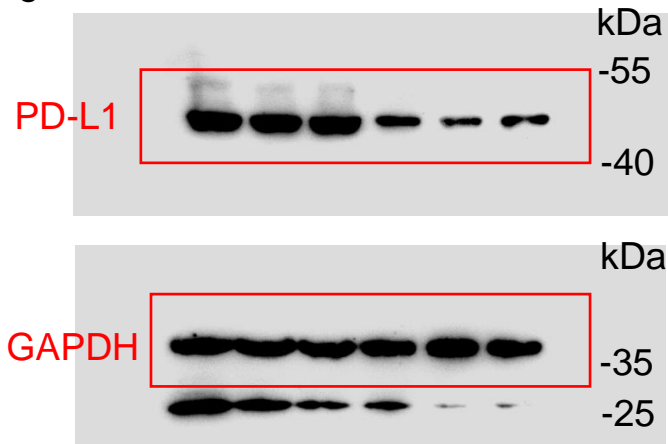

Figure 4q

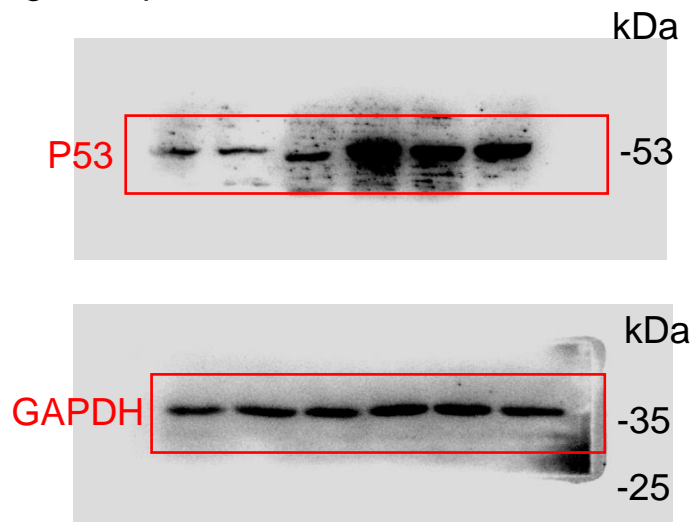

Figure 6n

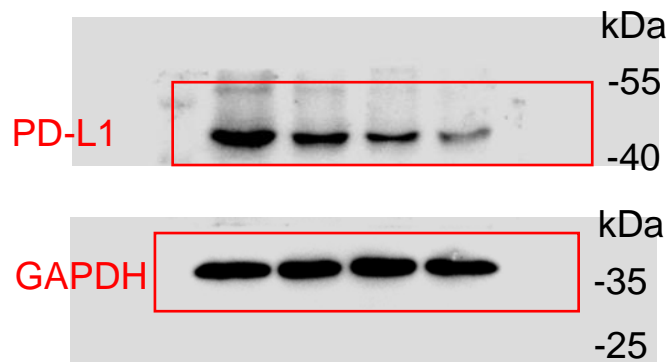

Supplement: Supplementary file 2 — Supplementary Material 2 [file 12885_2024_12224_MOESM2_ESM.pdf]
